# *Porphyromonas gingivalis* and its CRISPR-Cas system

**DOI:** 10.1080/20002297.2019.1638196

**Published:** 2019-07-03

**Authors:** Tsute Chen, Ingar Olsen

**Affiliations:** a Department of Microbiology, The Forsyth Institute, Cambridge, MA, USA; b Department of Oral Medicine, Infection, and Immunity, Harvard School of Dental Medicine, Boston, MA, USA; c Department of Oral Biology, Faculty of Dentistry, University of Oslo, Oslo, Norway

**Keywords:** CRISPR-Cas, *Porphyromonas gingivalis*, diversification, adaptive immunity, periodontitis, periodontal pocket, bacteriophages

## Abstract

The clustered regularly interspaced short palindromic repeats (CRISPRs) and their associated proteins (Cas) are immune systems in prokaryotes present in most Bacteria and Archaea. They provide adaptive immunity against foreign elements such as bacteriophages/viruses, plasmids and transposons. During immunization a small sequence of foreign DNA, a so-called spacer is integrated into the CRISPR locus in the host cell. Spacers are then transcribed into small RNA guides that direct cleavage of foreign DNA by Cas nucleases. Immunization through spacer acquisition is transferred vertically to the progeny. It is possible that this genetic immune system of bacteria participates in modulating the microbiome of ‘chronic’ periodontitis, in which *Porphyromonas gingivalis* has been identified as a keystone pathogen causing microbial dysbiosis. An in-depth review of our current knowledge on the CRISPR-Cas systems in *P. gingivalis* is given in this paper with the attempt to understand how this anaerobic bacterium may protect itself in the periodontal pocket where bacteriophages are abundant and even out-number bacteria.

Clustered regularly interspaced short palindromic repeats CRISPRs belong to a family of DNA sequences characterized by short direct repeats (DRs) that are separated by spacers [1]. Almost half (45%) of the species in Bacteria and the majority (80%) in Archaea contain CRISPR-Cas systems [2]. Through an adaptation process, the system is able to store memories of previous exposure to foreign DNA in the spacer sequences across generations of progenies [3–5]. Upon invasion of the same foreign DNA to the ‘immunized’ cells, the spacer sequences are transcribed into small CRISPR RNAs (crRNAs) which was used by the Cas proteins as a guide to cleave the foreign DNAs through reverse sequence complementarity [4,6]. Hence, the spacers serve as the ‘acquired memory’ of this adaptive immunization system and are at the center of CRISPR defense providing immunity against specific phages or plasmids. Cas proteins are encoded by the *cas* genes that are located proximal to the CRISPR array. The mechanism of the CRISPR-Cas system was first demonstrated in 2007 in *Streptococcus thermophilus* [7], a bacterium widely used in the dairy industry to make yogurt and cheese. The Cas protein that is being used for genome editing was engineered from the *cas9* gene of *S. pyogenes*, which is a pathogen that causes group A streptococcal (GAS) infection in humans [8].

CRISPR-Cas is a defense system commonly used by bacteria to fight phages/viruses, plasmids, transposons, integrative conjugative elements and genomic islands [2,9,10]. It is the only adaptive immune system in prokaryotes recognized so far. The system is also promising for programmable targeting of undesirable bacteria in microbial consortia. Interestingly, a significant association has been found between absence of CRISPR-Cas and the presence of antibiotic resistance [11]. *Porphyromonas gingivalis* is a Gram-negative anaerobic rod widely held as a keystone pathogen in ‘chronic’ periodontitis where it causes microbial dysbiosis [12,13]. It has also been associated with systemic diseases, e.g. cardiovascular and respiratory diseases, rheumatoid arthritis, premature birth and Alzheimer’s disease [14,15]. Almost 95% of clinical strains of *P. gingivalis* has been found to harbor CRISPR arrays [2]. The oral cavity is a frequent site for bacteriophages [16]. The vast majority of viruses detected in dental plaque has been identified as bacteriophages with only a few eukaryotic viruses such as herpesviruses and circoviruses present [17]. Bacteriophages may be active pathogens [16]. This was supported by Lum et al. [18] who found that many of the CRISPR loci in the mouth were transcribed and matched oral phages.

The aim of this review is to answer the question, to what extent *P. gingivalis*, recovered from periodontitis-affected sites and other locations, is equipped with CRISPR-Cas systems and how the CRISPR-Cas may function to protect *P. gingivalis*. An overview of most of the CRISPR-Cas systems studied so far in *P. gingivalis* will be given. A broader survey of the CRISPR-Cas systems in the genus *Porphyromonas* is also being conducted and the results will be presented elsewhere.

## Diversity, classification and evolution of CRISPR-Cas systems in bacteria and archaea

There is considerable diversity in the CRISPR-Cas systems of bacteria and archaea with respect to protein composition, effector complex structure, genome locus architecture, mechanisms of adaptation, pre-CRISPR (cr)RNA processing and interference [19]. The CRISPR-Cas system includes two main classes and six different types [20–22]. It has multi-subunit effector complexes in Class 1 and single protein modules in Class 2 [19]. In Class 2, two new types and several subtypes were recently identified [19]. The type VI systems were the first among CRISPR-Cas variants to exclusively target RNA. Also, in some of the Class 2 systems, the effector protein was additionally responsible for the pre-crRNA processing. It was suggested that Class 2 systems had evolved from mobile genetic elements on multiple, independent occasions.

## Classes and types of the CRISPR-Cas system in *P. gingivalis*

In *P. gingivalis* two different classes and four types of Cas systems were detected in 19 *P. gingivalis* genomes examined, based on the presence of different signature genes [23]. Watanabe et al. [24] detected six CRISPR/Cas types classified by sequence similarity of repeats in 13 *P. gingivalis* strains and 12 other types in 46 other *Porphyromonas* species. The CRISPR spacers with potential targets in the genus *Porphyromonas* were approximately 23 times more abundant than those with possible targets in other genera (1,720/6,896 spacers vs. 74/6,896 spacers) [25]. *Porphyromonas* CRISPR/Cas may therefore act by selective interference against intra- and interspecies nucleic acids as part of its genome plasticity.

## CRISPR structure and operons in *P.gingivalis*

**Figure 1.**
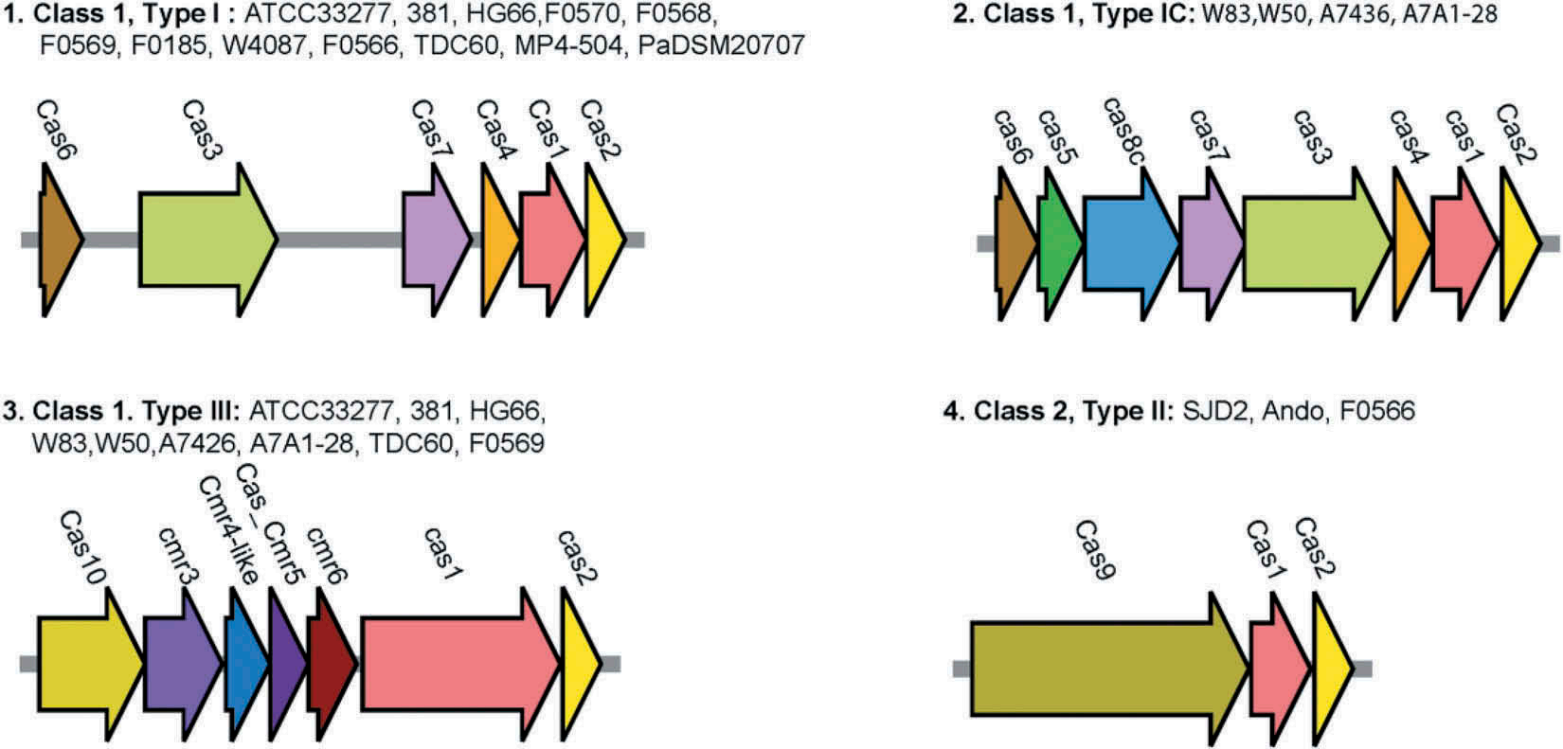
Four types of CRISPR-Cas systems detected by us in *P. gingivalis.*

Detailed structure of all the CRISPRs detected in 19 *P. gingivalis* genomes [23] is given in Table 1. To more comprehensively reveal the Cas proteins rather than relying on the somewhat incomplete default protein annotation provided by NCBI, all the protein sequences from the 19 *P. gingivalis* genomes were searched against a set of known Cas proteins to identify potential homologues. A total of 217,660 Cas proteins were downloaded from NCBI and used to search for the Cas homologues in *P. gingivalis*. This gave a complete picture of the Cas proteins in all *P. gingivalis* genomes that had been examined at that time. In addition, a few more CRISPR-associated genes were detected in *P. gingivalis* compared to those reported previously [23]. Figure 1 depicts the *cas* gene operons of the four types of CRISPR systems detected in *P. gingivalis*. The operon structure of each type of CRISPR systems is quite conserved in this species.

**Table 1.** Detailed structure of all the CRISPRs detected in 19 *P. gingivalis* genomes (from ref [23]).

Genome	Number of CRISPRs detected	DR_size(bps)*number of DRs
ATCC33277	3	36bps*13; 36bps*5; 30bps*120
HG66	3	30bps*97; 36bps*11; 37bps*6
381	3	36bps*11; 36bps*5; 30bps*120
W83	4	36bps*8; 36bps*8; 37bps*8; 30bps*24
W50	5	36bps*6; 36bps*8; 30bps*24; 37bps*4; 37bps*8
A7436	5	35bps*6; 36bps*8; 37bps*7; 37bps*5; 30bps*17
AJW4	2	46bps*4; 36bps*12
F0570	3	37bps*4; 30bps*6; 36bps*9
JCVI SC001	3	31bps*4; 36bps*6; 46bps*5
SJD2	3	36bps*9; 36bps*5; 46bps*22
F0568	7	30bps*4; 30bps*35; 30bps*10; 30bps*16; 36bps*6; 32bps*5; 32bps*5
F0569	22	30bps*8; 30bps*11; 30bps*5; 30bps*6; 30bps*5; 30bps*4; 30bps*4; 30bps*4; 30bps*5; 30bps*8; 30bps*4; 30bps*13; 30bps*10; 30bps*4; 30bps*8; 30bps*4; 30bps*8; 30bps*5; 33bps*4; 36bps*4; 36bps*5; 46bps*5
Ando	3	47bps*15; 36bps*4; 36bps*16
F0185	15	36bps*9; 27bps*6; 27bps*5; 31bps*4; 31bps*4; 31bps*5; 31bps*4; 31bps*4; 31bps*13; 31bps*4; 31bps*5; 31bps*6; 31bps*4; 31bps*4; 31bps*5
W4087	4	30bps*19; 36bps*7; 36bps*4; 36bps*5
MP4-504	3	36bps*6; 36bps*13; 30bps*37
A7A1-28	4	36bps*7; 30bps*26; 30bps*64; 37bps*9
F0566	7	32bps*5; 32bps*4; 30bps*11; 30bps*19; 46bps*4; 46bps*4; 46bps*10
TDC60	5	37bps*5; 30bps*17; 30bps*66; 30bps*16; 36bps*4
PaDSM20707	2	33bps*30; 23bps*4

## High diversity of CRISPR spacer contents in *P.gingivalis*

Preliminary examination of CRISPRs in three *P. gingivalis* strains (TDC60:89, W83:44 and ATCC33277:137) showed that CRISPR spacers with high nucleotide similarity to regions of *P. gingivalis* genomes were present and that the number of spacers was diverse among the three genomes [24]. In another 60 isolates of *P. gingivalis* the high diversity in CRISPR spacer contents was confirmed [25]. When the analyzed clusters were summed into four clusters, each type had distinct characters. The contents of the spacers differed amongst almost all the isolates, even if they were from the same patient. In another study the total number of spacers and unique spacers in *P. gingivalis* were 267/244 [26]. The 44 spacers in the genome of W83 had no significant similarity with any known sequences, but four spacers were similar to sequences of bacteria found in the oral cavity and gastrointestinal tract [2].

## *P. gingivalis* may selectively acquire DNA by CRISPRs for survival

Watanabe et al. [24] suggested that *P. gingivalis* might be a useful model to reveal the biological significance of CRISPR spacers with high nucleotide similarity to the genome of the same species; also, that *P. gingivalis* may selectively acquire useful DNA sequences for its own survival and evolution by the CRISPR function. They concluded that the CRISPR spacers in *P. gingivalis* possibly inhibit both genomic rearrangements and intercellular recombination among *P. gingivalis* strains. Furthermore, three *P. gingivalis* genomes harbored *cas* genes for DNA and RNA targeting. This supported the notion that CRISPRs in *P. gingivalis* may provide resistance to foreign RNA, as well as to DNA [20,27].

## CRISPRs may limit IS transposition and DNA uptake from other *P. gingivalis* cells

Watanabe et al. [24] also showed that *P. gingivalis* has high intraspecies diversity caused by frequent insertion sequence (IS) transposition. Seven spacers (7/19) revealed high nucleotide similarity to regions related to insertion segments in the genome of *P. gingivalis* [24]. CRISPRs may limit both IS transposition and the introduction of foreign DNA, mainly from other *P. gingivalis* cells.

## CRISPRS provide immunity against transposons

Transposase is an enzyme that binds to the two ends of a transposon and bring them together to form a loop. It then catalyzes movement of the transposon to another part of the genome by a cut and paste mechanism or replicative transposition. In the study by Chen et al. [23] who did comparative genomics of 19 *P. gingivalis* strains, a high prevalence of transposase proteins was found encoded in *P. gingivalis* (Table 2); actually as much as 149 copies in strain A7436. In another study transposases were found in all of the 35 genomes of *P. gingivalis* examined, varying in number from 8 (strains Ando, F0185, SDJ5) to 103 (A7436) [28]. The lower number of transposases detected in the original genomes that were not completely sequenced in this study was most likely due to the in-between-contig sequence gaps that may contain highly repeated sequences such as transposases and IS elements. The completed genome with the lowest number of mobility-related genes was that of strain A7A1-28, where 68 transposases were detected.

**Table 2.** Distribution of transposases (IS5 family; K07481) in 19 strains of *P. gingivalis.*

Annotation Source	ATCC33277	HG66	381	W83	W50	A7436	AJW4	F0570	JCVISC001	SJD2	F0568	F0569	Ando	F0185	W4087	MP4-504	A7A1-28	F0566	TDC60	PaDSM20707
KEGG Orthology	47	45	45	13	3	27	16	0	1	1	1	0	3	2	2	2	14	1	22	0

The number of proteins related to the IS5 transposase family was identified by the BlastKOALA program (ref [31]) with matching to the KEGG Orthology (KO) number K0748 (from ref [23]).

## *P. gingivalis* may limit genetic exchange in populations predisposed to exchange

Phillips et al. [29] found highly expressed transcripts of CRISPR regulatory small non-coding regulatory RNA (sRNA) in the intergenic sequences (IGS) upstream of CRISPR-associated (*cas*) gene arrays in *P. gingivalis* W83. Identification of these highly expressed sRNA transcripts indicated another mechanism that *P. gingivalis* uses to limit genetic exchange in a population with a genotype predisposed to a high degree of such exchange.

## Cas proteins do not necessarily follow CRISPRs in *P. gingivalis*

In the study by Chen et al. [23] CRISPR arrays were detected in all the 19 genomes examined, including the outgroup *Porphyromonas asaccharolytica* PaDSM20707 (Table 3). In contrast, Cas proteins were found only in select strains. Of 35 strains of *P. gingivalis* examined strain 13_1 encoded as many as 19 Cas homologous proteins and five different CRISPR systems were observed [28]. On the other hand, JSVI_ SC001, which is an environmental strain, contained no detectable proteins even though three CRISPR-like DNA sequence structures were detected.

**Table 3.** CRISPR arrays detected in 19 *P. gingivalis* genomes.

Annotation Source	ATCC33277	HG66	381	W83	W50	A7436	AJW4	F0570	JCVISC001	SJD2	F0568	F0569	Ando	F0185	W4087	MP4-504	A7A1-28	F0566	TDC60	PaDSM20707
NCBI	4	12	11	11	15	15	1	5	0	0	5	12	2	5	5	6	14	10	11	7
RAST	12	12	12	12	12	12	0	6	0	3	5	12	3	5	5	5	11	8	12	7
BLAST	**14**	**14**	**14**	**15**	**15**	**15**	**1**	**6**	**0**	**3**	**6**	**13**	**3**	**6**	**5**	**6**	**14**	**11**	**15**	**7**
CRISPR arrays	3	3	3	4	5	5	2	3	3	3	7	22	3	15	4	3	4	7	5	2

Results were compiled based on the NCBI or RAST genome annotations. Total number of proteins containing any of the keywords shown in each category was recorded for each genome and for NCBI and RAST annotations separately. BLAST: all the proteins identified by NCBI and RAST were collected and the sequences searched against all the proteins of all 20 genomes using BPLSTP. The numbers indicated for each genome are the number of proteins with ≥ 95% sequence identity and ≥ 95% coverage of the query sequences. The numbers were calculated separately for NCBI and RAST annotated proteins, and the larger number of the two is shown in this table. The number of CRISPR arrays was detected by the online software CRISPRfinger (http://crispr.i2bc.paris-saclay.fr/Server/); only the number of ‘confirmed’ candidates was reported thus excluding those ‘questionable’ ones, which only have two direct repeat elements (DRs), i.e. a type of genetic sequences that consist of two or more repeats of a specific sequence and one spacer sequences. (From ref [23])

Previously, the structure of the *cas* gene in *P. gingivalis* was reported to be conserved at two CRISPR loci, type 1-C and III-B [24]. By using the online software CRISPR finger (http://crispr.i2bc.paris-saclay.fr/Server) strain F0569 was found to have the highest number of CRISPR arrays (Table 3), but this strain did not have the highest number of Cas proteins [23]. The length of the direct repeat (DR) elements ranged from 23 to 47 bps among all the CRISPR arrays detected while the number of DRs in the arrays varied from five to 121 copies. The closely related strains ATCC33277 and 381 had three copies of almost identical CRISPR arrays with 121 DR sequences and 120 spacer sequences.

The number of CRISPR systems in the genome may indicate the difficulty in incorporating foreign DNA, whereas the number of DRs in the CRISPR elements may indicate CRISPR activity in the past for the genome. Zhou et al. [30] reported slightly higher Shannon-Wiener diversities of DRs in periodontal disease than in periodontal health, but the spacer composition exhibited significantly higher diversities in healthy periodontium. This may imply that healthy people have a robust and functional bacterial community able to resist invasion from phages. Strain JSVI_SC001 of *P. gingivalis*, isolated from single cells in the biofilm of a hospital bathroom sink, had three copies of CRISPR arrays with DRs of 31, 26 and 45 bps and repeat numbers of five, seven and six, respectively [23]. It might be that this strain had a type Cas protein clearly different from those in the oral strains. However, if this strain lacked any functional Cas protein, it could be susceptible to bacteriophage infection or activation by prophages possibly present, as indicated by the detection of 25 copies of phage-related proteins in this strain [23].

## Proteins related to reproduction of phage may be related to the presence of CRISPR/Cas systems in *P. gingivalis*

Many proteins related to reproduction of phage were detected in all the 19 strains of *P. gingivalis* examined [23]. This may help explain the abundant prevalence of CRISPR/Cas systems in this species.

## Functional activity of *P. gingivalis* CRISPRs detected *in vivo*

All four CRISPR regions in *P. gingivalis* W83 were found to be transcribed [2]. For one of the four arrays, activity was demonstrated *in vivo* against double-stranded DNA constructs that contained protospacer sequences accompanied by the 3ʹ end of an NGG protospacer-adjacent motif (PAM) (N is any nucleobase followed by two guanine (G) nucleobases). This was concluded from introduction of the flanked protospacer to foreign DNA, which caused specific degradation.

## CRISPR RNAs in the *P. gingivalis* CRISPR-Cas 1-C system

CRISPR RNAs (crRNAs) are important elements in the CRISPR-Cas system being responsible for the system’s selectivity and effectiveness [6]. crRNAs contain a spacer sequence flanked with 5ʹ and 3ʹ handles that originate from repeat sequences important for recognition of small RNAs by Cas. Burmistrz et al. [6] found that the 5ʹ handle of crRNA in *P. gingivalis* was essential for the activity while the repeat element localized to the 3ʹ handle was less important. In addition, modification of the structure of the repeat element inhibited its activity, possibly by interacting with the primary processing nuclease.

## Concluding remarks

The CRISPR/Cas system is known to prevent acquisition of conjugative plasmids, integrative conjugative elements and environmental DNA via natural transformation. To what extent this occurs in *P. gingivalis* is not yet clear. However, the widespread distribution of CRISPRs in *P. gingivalis* strains indicates that CRISPRs have an important role in this species. Interestingly, due to the fact that the spacer sequences derived more from its own genome, rather than foreign DNA, CRISPRs in this species may have developed as a selective system helping to regulate gene transfer. In contrast to CRISPRs, Cas proteins are only present in select *P. gingivalis* strains. The reason for this is also unclear. Previously, the structure of the *cas* gene in *P. gingivalis* was reported to be conserved at CRISPR loci. Most importantly, the CRISPR-Cas system of *P. gingivalis* has been found active *in vivo* providing protection against foreign genetic elements. This was supported by the observation that in most *P. gingivalis* genomes CRISPR-associated genes were found together as an operon and not scattered around the genome. More studies of the CRISPR systems in *P. gingivalis* both in wet laboratory and in-silico are needed to understand the true role of the CRISPR system in this periodontal pathogen and to what extent it is shaping the periodontal microbiome and the biological “arms race” occurring there. The CRISPR-Cas system may be involved in more functions than just the ‘immune’ system against the invader. A deeper understanding of this particular system may even shed more light on why *P. gingivalis* deserves the term keystone pathogen in periodontitis.
